# Hydatids of the Liver from a Camel

**Published:** 1883-07

**Authors:** 


### XIII.—HYDATIDS OF THE LIVER FROM A CAMEL.

REPORTED FROM THE COLLEGE.

A large camel from the Central Park was brought to the hospital almost in a state of collapse. No previous history was obtained. The animal was only in the building for a few hours.

At the necropsy the principal lesion worthy of note was found in the liver, which was filled with hydatid cysts, which resulted from the migration of the larva of the taenia ecchinococci. These cysts were in various states of retrograte change. Some were almost calcarious, some in a suppurative state, and some nearly cystic. The hooklets diagnostic of this lesion were easily demonstrated in great abundance at one of Prof. Satterthwaite's lectures.

